# Palmitoylethanolamide (PEA) as a Potential Therapeutic Agent in Alzheimer’s Disease

**DOI:** 10.3389/fphar.2019.00821

**Published:** 2019-07-24

**Authors:** Sarah Beggiato, Maria Cristina Tomasini, Luca Ferraro

**Affiliations:** ^1^Department of Life Sciences and Biotechnology, University of Ferrara, Ferrara, Italy; ^2^Technopole of Ferrara, LTTA Laboratory for the Technologies for Advanced Therapies, Ferrara, Italy; ^3^IRET Foundation, Bologna, Italy

**Keywords:** neuroinflammation, preclinical studies, animal models, 3xTg-AD, ultramicronized formulation

## Abstract

*N*-Palmitoylethanolamide (PEA) is a non-endocannabinoid lipid mediator belonging to the class of the *N*-acylethanolamine phospolipids and was firstly isolated from soy lecithin, egg yolk, and peanut meal. Either preclinical or clinical studies indicate that PEA is potentially useful in a wide range of therapeutic areas, including eczema, pain, and neurodegeneration. PEA-containing products are already licensed for use in humans as a nutraceutical, a food supplement, or a food for medical purposes, depending on the country. PEA is especially used in humans for its analgesic and anti-inflammatory properties and has demonstrated high safety and tolerability. Several preclinical *in vitro* and *in vivo* studies have proven that PEA can induce its biological effects by acting on several molecular targets in both central and peripheral nervous systems. These multiple mechanisms of action clearly differentiate PEA from classic anti-inflammatory drugs and are attributed to the compound that has quite unique anti(neuro)inflammatory properties. According to this view, preclinical studies indicate that PEA, especially in micronized or ultramicronized forms (i.e., formulations that maximize PEA bioavailability and efficacy), could be a potential therapeutic agent for the effective treatment of different pathologies characterized by neurodegeneration, (neuro)inflammation, and pain. In particular, the potential neuroprotective effects of PEA have been demonstrated in several experimental models of Alzheimer’s disease. Interestingly, a single-photon emission computed tomography (SPECT) case study reported that a mild cognitive impairment (MCI) patient, treated for 9 months with ultramicronized-PEA/luteolin, presented an improvement of cognitive performances. In the present review, we summarized the current preclinical and clinical evidence of PEA as a possible therapeutic agent in Alzheimer’s disease. The possible PEA neuroprotective mechanism(s) of action is also described.

## Introduction

Neuroinflammation and synaptic dysfunction in Alzheimer’s disease (AD) have been originally considered as epiphenomena with inflammation and altered neurotransmission occurring when damaged neurons provoke glia activation and changes in neuron biology. However, the growth of knowledge about the molecular mechanisms underlying AD converted this earlier view and points to a causal role of these events in the pathology (Overk and Masliah, 2014; Heneka et al., 2015; Steardo et al., 2015; Van Eldik et al., 2016; González-Reyes et al., 2017; Ahmad et al., 2019). Specifically, it is now well established that the pathogenesis of AD includes also interactions with immunological mechanisms/responses in the brain. Neuroinflammation in AD is predominantly linked to central nervous system (CNS)-resident microglia, astroglia, and perivascular macrophages, which have been implicated at the cellular level (Zádori et al., 2018). Regional inflammatory responses characterize the CNS in AD, with deposits of β-amyloid (Aβ) as foci, associated with increased expression of pro-inflammatory cytokines, acute phase proteins, and complement components, along with signs of activated microglia and reactive astrocytes (Skaper et al., 2018). According to this scenario, neuropathological studies in human brains, demonstrating the activation of glial cells, mainly microglia and astrocytes (Zimmer et al., 2014; Chaney et al., 2018; Edison et al., 2018; Knezevic and Mizrahi, 2018), have been corroborated by studies in animal models of AD in which an overproduction of pro-inflammatory signals by glial cells triggers a neurodegenerative cascade (Birch et al., 2014; Heneka et al., 2015; Chun et al., 2018; Saito and Saido, 2018). On the other hand, mounting evidence indicates that also oxidative stress and synaptic dysfunction are early events in AD (Overk and Masliah, 2014; Wirz et al., 2014; Kamat et al., 2016; Cai and Tammineni, 2017). Changes in neuronal activity/signaling in AD can promote the β-amyloidogenic pathway of amyloid precursor protein (APP) processing, leading to increased Aβ levels and thus creating a sort of a positive feedback or a vicious cycle to accelerate AD pathogenesis (Herrup, 2010; Wirz et al., 2014; Cai and Tammineni, 2017). These findings indicate that neuroinflammation, oxidative stress, and synaptic dysfunction are integral parts of AD pathogenesis, and not solely consequences of Aβ-induced CNS damage. Thus, the relationship between neurodegeneration and neuroinflammation is strictly interdependent, suggesting that compounds able to simultaneously target these processes might be effective therapeutic agents in AD. In this context, endocannabinoid signaling and endocannabinoid-related compounds have been demonstrated to modulate the main pathological processes during early AD, including protein misfolding, neuroinflammation, excitotoxicity, mitochondrial dysfunction, and oxidative stress (Aso and Ferrer, 2014; Bedse et al., 2015; Fernández-Ruiz et al., 2015). Among these compounds, *N*-palmitoylethanolamide (PEA) has attracted much attention because it exerts a local anti-injury function through a down-modulation of mast cells and protects neurons from excitotoxicity through several mechanisms (Mattace Raso et al., 2014; Petrosino and Di Marzo, 2017).

PEA is a non-endocannabinoid lipid mediator belonging to the class of the *N*-acylethanolamine (NAE) phospolipids, which also includes the first endocannabinoid to be discovered, *N*-arachidonoyl-ethanolamine (anandamide; AEA) and the anorectic mediator *N*-oleoyl-ethanolamine (OEA). PEA was firstly isolated from soy lecithin, egg yolk, and peanut meal (Ganley et al., 1958; Petrosino and Di Marzo, 2017). Either preclinical or clinical studies indicate that PEA is potentially useful in a wide range of therapeutic areas, including eczema, pain, and neurodegeneration. PEA-containing products (Normast^®^, Glialia^®^, Nevamast^®^, Adolene^®^, Visimast^®^, Mastocol^®^, and Pelvilen^®^) are already licensed for use in humans (generally 1,200 mg/day) as a nutraceutical, a food supplement, or a food for medical purposes, depending on the country. PEA is especially used in humans for its analgesic and anti-inflammatory properties (Petrosino and Di Marzo, 2017; Tsuboi et al., 2018) and has demonstrated high safety and tolerability (Gabrielsson et al., 2016; Nestmann, 2016; Petrosino and Di Marzo, 2017). In the last decade, several studies suggested that PEA might exert protection against neuroinflammation and neurodegeneration, thus indicating that the compound possesses exceptional potential as a novel treatment for neurodegenerative disorders (Hansen, 2010; Skaper et al., 2014; Iannotti et al., 2016; Brotini et al., 2017; D’orio et al., 2018; Scuderi et al., 2018).

In this review, we initially briefly discuss the main molecular targets of PEA and its pharmacological properties, including the available pharmacokinetic data. Successively, we report the *in vivo* and *in vitro* findings, along with clinical results, supporting the possible role of PEA as a therapeutic agent in AD.

## Pharmacology of PEA

**Figure 1:**
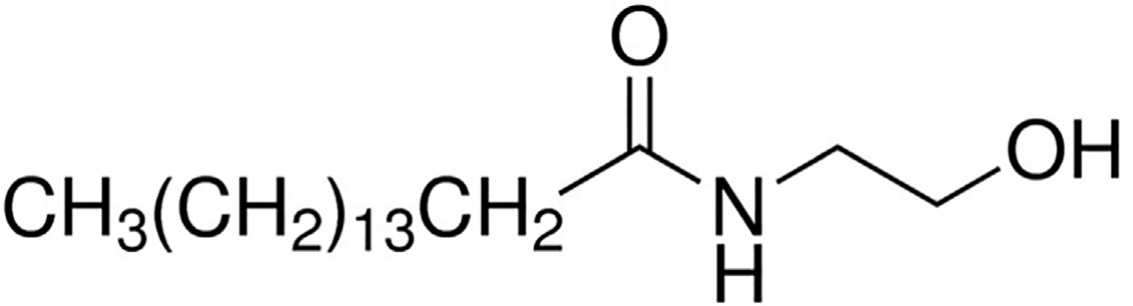
Chemical structure of palmitoylethanolamide.

PEA attracted the interest of the scientific community mainly after the discovery by an Italian Nobel Prize laureate Rita Levi Montalcini and co-workers that some acylethanolamides, initially termed ALIA-amides (autacoid local injury antagonist; ALIA) are endogenously synthesized lipids exerting interesting anti-inflammatory properties (Levi-Montalcini et al., 1996). PEA (C16:0 *N*-acylethanolamine; **Figure 1**) is a lipid mediator biologically synthetized in many plants as well as in cells and mammal tissues. It belongs to the class of non-endocannabinoid NAE, which also includes stearoylethanolamide (C18:0 *N*-acylethanolamine), oleoyl-ethanolamide (OEA, C18:1 *N*-acylethanolamine), and linoleoylethanolamide (C18:2 *N*-acylethanolamine). These compounds are much more abundant than the endocannabinoid anandamide in several animal tissues and endowed with important biological actions. The biosynthesis and metabolism of PEA have been deeply described elsewhere (Petrosino et al., 2010; Tsuboi et al., 2013; Petrosino and Di Marzo, 2017; Tsuboi et al., 2018), and we refer to those reviews for their description.

### Mechanisms of Action of PEA: Focus on Neuroinflammation

Several preclinical *in vitro* and *in vivo* studies have demonstrated that PEA can induce its biological effects by acting on several molecular targets in both central and peripheral nervous systems (Mattace Raso et al., 2014; Iannotti et al., 2016; Petrosino and Di Marzo, 2017; Tsuboi et al., 2018). As reported above, it has been initially suggested that PEA, belonging to the class of acylethanolamides, exerts its anti(neuro)inflammatory effects by acting as an “autacoid local injury antagonist” (ALIA) leading to a down-regulation of mast cell activation (Levi-Montalcini et al., 1996). However, subsequent preclinical studies strongly supported the view that PEA can directly activate at least two different receptors: the peroxisome proliferator-activated receptor-alpha (PPAR-α; Lo Verme et al., 2005) and the orphan GPCR 55 (GPR55; Pertwee, 2007).

PPAR-α actually seems to be the main molecular target involved in the anti(neuro)inflammatory effects of PEA. PPAR-α is, in fact, known for its protective role against (neuro)inflammation, and PPAR-α ligands are recognized as possible anti-inflammatory compounds (Devchand et al., 1996; Straus and Glass, 2007). When activated by a ligand, PPAR-α forms a heterodimer with 9-*cis*-retinoic acid receptor (RXR) able to interact with specific DNA sequences in the promoter regions of selective genes, thus leading to complex anti-inflammatory responses (Daynes and Jones, 2002). *In vitro*, PEA is able to activate PPAR-α with a half-maximal effective concentration (EC_50_) of 3.1 ± 0.4 μM (De Gregorio et al., 2018). Numerous studies demonstrated that PPAR-α antagonist or the genetic ablation of this receptor counteracts/prevents the protective effects of PEA against neuroinflammation and neurodegeneration in cellular or animal models of different pathologies (Scuderi et al., 2011; D’Agostino et al., 2012; Esposito et al., 2012; Paterniti et al., 2013a; Avagliano et al., 2016; Cristiano et al., 2018), thus supporting the relevance of this target in the mechanism of action of PEA.

PEA has shown agonist activity towards the orphan receptor GPR55 (Baker et al., 2006), which was proposed as a third cannabinoid receptor (Pertwee, 2007; Yang et al., 2016). In fact, cannabinoids are able to interact with GPR55, thus inducing some behavioral, immunological, and neuroinflammatory activities (De Gregorio et al., 2018; Balenga et al., 2014). However, the limited sequence similarity between GPR55 and cannabinoid receptors does not support this concept (Baker et al., 2006). At the present, the relevance of this receptor activation in the anti-inflammatory/neuroprotective PEA-induced effects remains to be clarified. It has been reported that PEA improves murine experimental colitis and that this effect is, at least partially, mediated by GPR55 activation (Borrelli et al., 2015). Furthermore, PEA protects against atherosclerosis by promoting an anti-inflammatory and proresolving phenotype of lesional macrophages, and this effect involves GPR55 activation (Rinne et al., 2018). The expression of GPR55 was protective against the insult exerted by MPP^+^ in a cellular model of Parkinson’s disease, but an agonist of GPR55 did not enhance neuroprotection in GPR55-expressing cells (Martínez-Pinilla et al., 2019). However, the GPR55 agonist abnormal-cannabidiol (Abn-CBD), a synthetic cannabidiol isomer, displayed beneficial properties when chronically administered (5 weeks) to a murine model of Parkinson’s disease (Celorrio et al., 2017). Moreover, a neuroprotective role of GPR55 activation on neural stem cells *in vitro* and *in vivo* has been recently proposed, thus suggesting that GPR55 could provide a novel therapeutic target against negative regulation of hippocampal neurogenesis by inflammatory insult (Hill et al., 2019). Finally, a selective agonist for GPR55 protected dentate gyrus granule cells and reduced the number of activated microglia after NMDA induced lesions in an *in vitro* model of rat organotypic hippocampal slice cultures (Kallendrusch et al., 2013). Taken together, these data suggest that the beneficial anti-neuroinflammatory effects of PEA might be mediated, at least in part, by GPR55 activation. However, other data suggested anti-inflammatory properties of GPR55 blockade. For example, a GPR55 antagonist diminished inflammation in experimental colitis by reducing the levels of pro-inflammatory cytokines, tumor necrosis factor-alpha (TNF-α), interleukin (IL)-1β, and IL-6 and impairing leukocyte activation and migration (Stančić et al., 2015). In addition, anti-neuroinflammatory effects have been observed after the treatment of LPS-activated primary microglial cells with a GPR55 inverse agonist (Saliba et al., 2018). Thus, the precise role of GPR55 in the anti-inflammatory/neuroprotective PEA action remains to be elucidated.

Besides its direct action on PPAR-α and GPR55, compelling evidence indicates that PEA could produce several indirect receptor-mediated actions, through the so-called entourage effect (Mattace Raso et al., 2014; Petrosino and Di Marzo, 2017). Given its weak affinity for CB1 and CB2 receptors, cannabinoid receptors are not considered direct targets of PEA. However, PEA can indirectly activate cannabinoid receptors through different indirect mechanisms. In particular, PEA may indirectly activate CB1 and CB2 receptors by acting as a false substrate for fatty acid amide hydrolase (FAAH), the enzyme involved in the degradation of the endocannabinoid AEA (Petrosino et al., 2016; Petrosino and Di Marzo, 2017), thus leading to a reduced degradation of AEA. This action leads to increased levels of AEA and, in turn, an increased activation of cannabinoid receptor-mediated signaling. Furthermore, quite recent studies have demonstrated that PEA increases the levels of CB2 receptor mRNA and protein as a result of PPAR-α activation, and this effect is involved in PEA-induced microglia changes associated with increased migration and phagocytic activity (Guida et al., 2017). Finally, the discovery that GPR55 forms receptor heteromer with either CB1 or CB2 receptors (Balenga et al., 2014; Martínez-Pinilla et al., 2014; Martínez-Pinilla et al., 2019) raises the exciting possibility that PEA might modulate CB1- and/or CB2-mediated intracellular signaling by targeting the GPR55 protomer in these putative GPR55/CB1 or GPR55/CB2 heterodimers. PEA can also indirectly activate the transient receptor potential vanilloid type 1 (TRPV1) channel, which is also a target for the endocannabinoids (Zygmunt et al., 2013), *via* different mechanisms. In particular, PEA-induced increase of endocannabinoid levels can modulate inflammation and other immune functions *via* TRPV1 channel (Ross, 2003). In addition, putative allosteric properties of PEA at TRPV1 channels have been proposed to possibly explain the ability of the compound to increase the endocannabinoid-induced activation and desensitization of TRPV1 channels (Petrosino and Di Marzo, 2017). Finally, as the existence of a direct biochemical interaction has been proposed, it seems likely that PEA can also indirectly activate TRPV1 channels *via* PPAR-α activation. The possible relevance of these mechanisms in the anti-neuroinflammatory/neuroprotective effects of PEA remains to be clarified. In fact, TRPV1 channel activation has been linked to either anti-neuroinflammatory or pro-neuroinflammatory signaling (Kong et al., 2017). Interestingly, it has been recently reported that TRPV1 activation reduces central inflammation in multiple sclerosis (Stampanoni Bassi et al., 2019). Accordingly, neuroprotective effects of TRPV1 activation in animal models of Parkinson’s and Alzheimer’s diseases have been reported (Jiang et al., 2013; Nam et al., 2015; Jayant et al., 2016; Xu et al., 2017; Zhao et al., 2017; Balleza-Tapia et al., 2018).

Taken together, the above findings strongly suggest that PEA by activating multifactorial pharmacological targets and by mediating several cellular mediators could play promising protective roles in contrasting neuroinflammation and neurodegeneration. The ability of PEA to synergistically interact *via* several mechanisms is attributed to the compound’s quite unique properties in respect to the traditional anti-inflammatory drugs.

### Pharmacokinetic

Given its poor water solubility, large particle size in the native state, and, possibly, short‐lived action, PEA might have limitations in terms of solubility and bioavailability. In fact, PEA is almost insoluble in water, while its solubility in most other aqueous solvents is very poor with a partition coefficient (log *P*) estimated to be > 5 (Lambert et al., 2001). Published data on PEA bioavailability are still scarce, but recent findings are contributing to better understand the pharmacokinetic of the compound and the possible relevance of new oral formulations.

It was originally reported that in rats, following its intraperitoneal (i.p.) administration, *N*‐[1‐^14^C]‐PEA was mainly distributed in some peripheral organs, and the lower concentrations were found in the brain, plasma, and erythrocytes (Zhukov, 1999). Moreover, orally administered *N*‐[9,10‐^3^H]‐PEA (100 mg/kg of body weight) was able to penetrate through the blood–brain barrier (BBB), but only in small amounts with a brain bioavailability corresponding to 0.95% of the oral dose (Artamonov et al., 2005; Gabrielsson et al., 2016). It has also been reported that PEA administration to humans leads to a two- to nine-fold increase in plasma baseline concentrations, depending on the dose (Balvers et al., 2013). The poor pharmacokinetic of PEA prompted the development of different formulation strategies, especially aimed at ameliorating the compound distribution. For instance, it has been demonstrated that when PEA was formulated as an emulsion in corn oil and administered subcutaneously (s.c.) to young DBA/2 mice (10 mg/kg of body weight), the compound was more extensively distributed in several organs, including the brain (Grillo et al., 2013).

In addition, a PEA suspension in corn oil administered to rats by gastric gavage (100 mg/kg of body weight) led to an about 20-fold increase in basal PEA plasma levels (Vacondio et al., 2015). The highest PEA plasma concentration was observed after 15 min (*C*_max_ = 420 ± 132 nM); PEA plasma levels returned to the baseline ones ∼2 h after the compound administration. The formulation of PEA as micronized or ultramicronized particles (m‐PEA and µm‐PEA, respectively) has been more recently proposed as a strategy to possibly increase PEA bioavailability, also in the CNS, without affecting the pharmacological efficacy of the compound (Impellizzeri et al., 2014; Petrosino and Di Marzo, 2017; Petrosino et al., 2018). It has been firstly reported that the oral administration of µm‐PEA (30 mg/kg of body weight) to a beagle dog led to a five-fold increase in blood PEA concentration. The peak of plasma PEA levels (∼55–60 pmol/ml) was observed 1 and 2 h after the administration of the compound (Cerrato et al., 2012). Subsequently, another study confirmed this finding (Petrosino et al., 2016). Another pharmacokinetic profile of m‐PEA and µm‐PEA after a single oral administration (15 mg/kg of body weight) to beagle dogs is reported in a US patent (Della Valle et al., 2013). In this case, blood samples have been taken at time 0 (immediately before the administration of PEA) and at times (*t*) 1, 2, and 3 h; the administration of m‐PEA and µm‐PEA leads to similar peak concentration values of PEA in serum (22.2 and 22.4 pmol/ml, respectively; ∼2 times higher than the baseline values) measured in the blood samples taken 1 h after the compound administration, with PEA concentrations returning to basal values at *t* = 2 h. Petrosino et al. (2016) reported the first preliminary pharmacokinetic data in humans. In particular, the authors measured blood PEA concentrations after the oral administration of m-PEA (300 mg) to human volunteers. Blood sample collection was carried out immediately before, and after 2, 4, and 6 h after PEA assumption; under this conditions, the peak of plasma PEA levels (∼22 pmol/ml) was observed 2 h after the compound assumption, with a drop to baseline levels within the following 2 h. Very recently, Petrosino et al. (2018) demonstrated by orally administering µm‐[^13^C]4-PEA or a naïve [^13^C]4-PEA (30 mg/kg of body weight) formulation to healthy and carrageenan-injected rats, that ultramicronization increases the ability of PEA to reach peripheral and central tissues under either healthy or local inflammatory conditions. In particular, the plasma concentrations of [^13^C]4-PEA were measured at 5, 15, 30, and 60 min after the oral administration of the compound in ultramicronized and naïve formulations to healthy rats. Rats receiving µm-[^13^C]4-PEA showed higher mean plasma levels of the compound than rats receiving naïve [^13^C]4-PEA. In rats receiving µm‐[^13^C]4-PEA, the peak concentration of [^13^C]4-PEA (5.4 ± 1.87 pmol/ml) was found after 5 min, and it was five times higher than the concentration measured in rats administered with the naïve formulation (1.1 ± 0.35 pmol/ml), in which no significant peak plasma concentrations were found.

Collectively, the above findings suggest that micronized or ultramicronized formulations of PEA maximize the compound bioavailability and efficacy, although further studies are necessary to undoubtedly confirm this hypothesis. Other strategies have been proposed to improve PEA bioavailability. For instance, PEA ester derivatives, prepared by conjugating PEA with various amino acids, have been synthetized as PEA prodrug and allowed to modulate the kinetics of PEA release in plasma and stability in liver homogenate (Vacondio et al., 2015). Two derivatives, l-Val-PEA, with suitable PEA release in plasma, and d-Val-PEA, with high resistance to hepatic degradation, were orally administered to rats, and plasma levels of prodrugs and PEA were measured at different time points, in comparison with naïve PEA (equimolar doses corresponding to 100 mg/kg of PEA). Both prodrugs showed significant release of PEA but provided lower plasma concentrations than those obtained with equimolar doses of naïve PEA. The highest PEA plasma concentrations were observed after 15 min following PEA, l-Val-PEA, or d-Val-PEA (420 ± 132, 56.4 ± 13.5, or 53.9 ± 19.7 nM, respectively). It has also been reported that the loading of the compound in nanostructured lipid carriers (NLCs) enhances the ocular bioavailability of PEA (Puglia et al., 2018) and that polyethylene glycol esters of PEA proved to be able to delay and prolong the pharmacological activity of the compound (Tronino et al., 2015), thus suggesting that these formulations might also ameliorate systemic PEA pharmacokinetic.

## PEA and Alzheimer’s Disease

Several preclinical and some clinical indications support the view of PEA as a therapeutic tool with high potential for the effective treatment of different pathologies characterized by neurodegeneration and neuroinflammation (Calabrò et al., 2016; Brotini et al., 2017; Holubiec et al., 2018; Impellizzeri et al., 2019). In this context, the potential beneficial effects of PEA have been demonstrated in several *in vitro* and *in vivo* experimental models of AD.

### Preclinical Evidence

#### *In Vitro* Studies

To our knowledge, the first experimental indication of PEA as a possible therapeutic agent in AD has been published by Scuderi et al. (2011). In their pioneering work, the authors evaluated the ability of PEA (10^−7^ M) to mitigate Aβ (Aβ_1–42_; 1 μg/ml)-induced astrogliosis in primary cultures of rat astrocytes. The results indicate that PEA treatment attenuated Aβ-induced astrocyte activation, as proven by its effects in reducing astrocyte hypertrophied cell bodies and thickened processes, along with the expression of glial fibrillary acidic protein (GFAP) and S100 calcium-binding protein B (S100B), two specific markers of astrocyte activity also linked to AD pathogenesis. Furthermore, PEA was able to blunt Aβ-induced neuroinflammation by significantly diminishing either the altered expression of pro-inflammatory molecules, such as cyclooxygenase-2 (COX-2) and inducible nitric oxide synthase (iNOS), or the enhanced release of prostaglandin PGE2, nitric oxide, IL-1β, and TNF-α. Interestingly, the PPAR-α antagonist MK886 was able to partly blunt the PEA-induced effects against Aβ-induced astrogliosis and neuroinflammation, thus suggesting a significant, but not exclusive, involvement of the PPAR-α in mediating the above-mentioned PEA actions. Concerning the possible intracellular signaling involved in PEA-induced effects, it has also been demonstrated that PEA critically diminished the Aβ-induced activation of p38 and Jun N-terminal kinase (JNK), as well as the subsequent activation of nuclear transcription factors, such as nuclear factor kappaB (NF-kB) and activator protein 1 (AP-1) (Scuderi et al., 2011). Later, the same group demonstrated that PEA treatment exerted protective effects against Aβ-induced toxicity also in primary rat mixed neuroglial co-cultures and organotypic hippocampal slices (Scuderi et al., 2012; Scuderi and Steardo, 2013). In particular, in mixed neuroglial co-cultures PEA prevented the increase in astrocyte number and the quantity of apoptotic nuclei in microtubule-associated protein 2 (MAP2)-positive neurons induced by Aβ challenge. Under these experimental conditions, the PEA antigliosis and neuroprotective effects were completely ascribed to PPARα activation, since MK886, the selective PPARα antagonist, almost completely abolished the PEA-induced effects. On the contrary, GW9662, a selective PPARγ antagonist, did not exert any significant influence. Furthermore, PEA decreased Aβ-induced astrocyte and microglia activation in organotypic cultures of rat hippocampi, an effect associated with a rescue of neuronal CA3 damage caused by Aβ challenge. PEA treatment also rescued neuron integrity and reduced the levels of neuroinflammation markers in this preparation. Once more, these effects were completely abolished by the pretreatment with a PPARα antagonist (Scuderi and Steardo, 2013). Finally, PEA was also evaluated for its possible effects in AD angiogenesis and neuroinflammation by using Aβ-treated C6 rat astroglioma cells and human umbelical vein endothelial cells (HUVEC)(Cipriano et al., 2015). In line with the previous findings, under these experimental conditions, PEA concentration-dependently reduced the expression of pro-inflammatory and pro-angiogenic markers in Aβ (1 μg/ml)-stimulated C6 cells. Interestingly, the medium aspired from PEA-treated C6 cells was able to reduce the HUVEC proliferation induced by their exposure to the conditioned medium from Aβ-treated C6 cells. The possible anti-angiogenic properties of PEA were also supported by the demonstration that the compound inhibited the nuclear levels of mitogen-activated protein kinase 1, which is associated with the main pro-angiogenic pathway, as well as the cytoplasmic vascular endothelial growth factor in HUVEC exposed to the medium from Aβ-treated C6 rat astroglioma cells. Once again, these effects were blocked by the treatment with the PPAR-α antagonist GW6471. As the release of proangiogenic factors during astrogliosis has been suggested as a key step in controlling AD progression, these findings further support the role of PEA as therapeutic agents for AD (Cipriano et al., 2015).

During the last years, other groups confirmed the protective action of PEA against the *in vitro* toxic effects of Aβ. For instance, in a very elegant study, it has been demonstrated that in wild-type (WT) mice, the addition of several acylethanolamides (including PEA) partially reverted Aβ-induced inflammation. However, the genetic deletion of FAAH (i.e., the enzyme involved in the degradation of the endocannabinoid AEA) in astrocytes induced an increased sensitivity to the pro-inflammatory Aβ-induced action, and this effect involved PPAR-α, PPAR-γ, and TRPV1 receptors, but not CB_1_ or CB_2_ receptors (Benito et al., 2012). Based on these findings, the authors raised the possibility that an excessively prolonged enhancement of the endocannabinoid tone may have harmful consequences, instead of the beneficial effects exerted by an acute increased tone.

Moving from the above data, we evaluated the protective role of PEA against Aβ-induced toxicity on cell viability and glutamatergic transmission in primary cultures of cerebral cortex neurons and astrocytes from the triple-transgenic murine model of AD (3xTg-AD) and their WT littermates (non-Tg mice; Tomasini et al., 2015). 3xTg-AD mice were selected because these animals harbor three mutant human genes (APP_Swe_, PS1_M146V_, and tau_P301L_) and closely mimic many aspects of AD in humans. In fact, these animals are characterized by age-dependent build-up of both plaques and tangles in the cerebral cortex, hippocampus, and amygdala regions, along with early synaptic dysfunction and cognitive decline, thus constituting a widely used and validated AD model. The results indicated that Aβ_1-42_ fragment (0.5 μM; 24 h) treatment induced a reduction of cell viability and an increase in glutamate levels in cultured cortical neurons and astrocytes from non-Tg mice, but not in those from the genetic model of AD. The Aβ-induced effects in non-Tg cell cultures were counteracted by a pretreatment with PEA (0.1 μM). Based on these findings, it has been hypothesized that exogenous Aβ treatment failed to induce deleterious effects in 3xTg-AD mice-derived cortical neurons, as these cells at 8 days *in vitro* were already exposed to a quite high concentration of endogenous peptide fragment. In fact, Aβ levels were observed in these cell cultures after 6 days *in vitro* (Vale et al., 2010), and we demonstrated that control cultured cortical neurons obtained from 3xTg-AD mice displayed morphological alterations similar to those observed in Aβ-exposed cultured cortical neurons obtained from non-Tg mice (Tomasini et al., 2015). However, the treatment with PEA prevented the effects of Aβ in cultured cortical neurons and astrocytes from non-Tg mice but failed to affect the morphological alterations and glutamate levels in 3xTg-AD mice-derived cell cultures. This suggests that the compound may be effective in the early AD or when Aβ is accumulating, thus initiating to damage the CNS. Later, Bronzuoli et al. (2018), by using a different *in vitro* protocol, demonstrated that PEA did not display toxic effects in both astrocytes and neurons from 3xTg-AD mice, at the tested concentrations (0.01, 0.1, and 1 μM), but it promoted neuron viability and counteracted reactive astrogliosis in mature 3xTg-AD primary astrocytes.

In a further study, we evaluated whether astrocytes could participate in regulating the Aβ-induced neuronal damage, by using primary mouse astrocyte cell cultures and mixed astrocyte–neuron cultures (Beggiato et al., 2018). The results indicated that in the presence of astrocytes pre-exposed to Aβ_1-42_ fragment (0.5 μM; 24 h), there was a reduction of neuronal viability, an increase in the number of neuronal apoptotic nuclei, a decrease in the number of MAP-2-positive neurons, and an increase in the number of neurite aggregations/100 μm as compared with control (i.e., untreated) astrocyte–neuron co-cultures. Taken together, these data indicate that astrocytes contribute to Aβ-induced neurotoxicity and neuroinflammation. Interestingly, these effects were not observed when neurons were cultured in the presence of astrocytes pre-exposed to PEA (0.1 μM), applied 1 h before and maintained during Aβ treatment. Thus, it has been concluded that PEA, by blunting Aβ-induced astrocyte activation, improved neuronal survival in mouse astrocyte–neuron co-cultures.

Finally, other researchers investigated the possible anti-AD action of co-ultraPEALut, a co-ultramicronized formulation of PEA in combination with the vegetable flavonoid luteolin (Lut), which demonstrated antioxidant properties. Previous studies, in fact, indicated that the association of these two molecules, in a fixed ratio of 10:1 in mass, induced a strong neuroprotective activity (Paterniti et al., 2013b). The exposure of human neuronal cells obtained by differentiating SH-SY5Y neuroblastoma cells to Aβ_1-42_ (1 µM; 24 h) induced a reduction of cell viability and neuroinflammatory responses. These effects were counteracted by the pre-treatment with co-ultraPEALut (reference concentrations: 27, 2.7, and 0.27 µM OF PEA) for 2 h (Paterniti et al., 2014). Similar results were obtained from an *ex vivo* organotypic model of AD. In particular, hippocampal slice cultures were prepared from mice at postnatal day 6, and after 21 days of culturing, the slices were pre-treated with co-ultraPEALut and then incubated with Aβ_1-42_ fragment (1 µM; 24 h). Under these experimental conditions, the pre-treatment with co-ultraPEALut significantly reduced iNOS and GFAP expression, restored neuronal iNOS and brain-derived neurotrophic factor (BDNF), and reduced the apoptosis (Paterniti et al., 2014). In line with these data, co-ultraPEALut reduced the expression of mRNA codifying serum amyloid A (SAA) in oligodendrocyte precursor cells subjected to TNF-α treatment. The relevance of this finding is supported by the evidence that SAA immunoreactivity is found in axonal myelin sheaths of cortex in AD (Barbierato et al., 2017).

#### *In Vivo* Studies

The promising *in vitro* results prompted the development of *in vivo* studies aimed at evaluating the neuroprotective properties of PEA in animal models of AD. Firstly, D’Agostino et al. (2012) tested PEA against the learning and memory dysfunctions induced in mice by the intracerebroventricular injection of Aβ_25–35_ peptide (9 nmol). To this purpose, PEA was administered once a day (3–30 mg/kg, s.c.), starting 3 h after Aβ_25–35_, for 1 or 2 weeks, while water-maze, water-maze working memory, and novel object recognition tests were used to assess cognitive performances. The authors demonstrated that, depending on the dose, PEA reduced (10 mg/kg of body weight) or prevented (30 mg/kg of body weight) the cognitive impairments induced by Aβ_25–35_ peptide injection. In line with previous *in vitro* findings, the beneficial effects of PEA appear mediated by PPAR-α, as the compound failed to rescue memory deficits induced by Aβ_25–35_ peptide injection in PPAR-α null mice, and GW7647 (a synthetic PPAR-α agonist) mirrored the effects of PEA. These encouraging behavioral results were corroborated by the evidence that in the same animals used for cognitive tests, PEA reduced brain lipid peroxidation, protein nitrosylation, iNOS induction, and caspase3 activation (D’Agostino et al., 2012). Comparable results have been obtained following the intrahippocampal injection of Aβ_1–42_ combined with PEA treatment in adult male rats (Scuderi et al., 2014). Immunofluorescence analysis of the hippocampal CA3 area ipsilateral to the injection site revealed that injection of Aβ_1–42_ induced astrocyte activation, as demonstrated by the fact that these cells showed a stellate shape and multiple branched processes. An increased expression of GFAP and S100B mRNA and protein, as well as increased densities of S100B-positive astrocytes, was also observed. Finally, intrahippocampal injection of Aβ_1–42_ was also associated with an upregulation of inflammatory markers, such as iNOS, COX-2, IL-1β, and TNF-α in homogenates of hippocampi ipsilateral to the injection site. When PEA (10 mg/kg of body weight) was intraperitoneally administered once a day for seven consecutive days, starting from the day of Aβ_1–42_ injection, it was able to partially or completely antagonize Aβ_1–42_-induced toxic effects. Again, the effects of PEA were prevented by the treatment with GW6471 (2 mg/kg), thus demonstrating the involvement of PPAR-α. Moreover, the authors demonstrated the PPAR-α-dependent ability of PEA to restore the alteration in the Wnt signaling pathway caused by Aβ_1–42_ hippocampal infusion. This is relevant since Wnt signaling pathway plays different roles in the development of neuronal circuits and also in the adult brain, where it regulates synaptic transmission and plasticity and has been also implicated in various diseases including neurodegenerative diseases (Inestrosa and Varela-Nallar, 2014). Finally, PEA reduced phosphorylated tau protein overexpression and rescued cognitive functioning, further strengthening the potential properties of the compound as a therapeutic agent in AD (Scuderi et al., 2014).

More recently, *in vivo* studies demonstrated that PEA displays beneficial effects also in a genetic model of the pathology. In a first study, the effects of chronic administration (3-month treatment) of µm‐PEA in 3xTg-AD mice, at two different stages of the pathology, were evaluated by administering the compound *via* a subcutaneous delivery system to groups of 3-month-old and 9-month-old animals (Scuderi et al., 2018). The animals were then tested at the end of the 3-month treatment and thereafter at the age of 6 months (i.e., early-symptomatic stage) and 12 months (i.e., clearly symptomatic stage), respectively. A battery of cognitive and non-cognitive tasks, followed by biochemical assessments of neuropathology, have been performed. Under these experimental conditions, µm‐PEA rescued cognitive functions in 6-month-old 3xTg-AD mice as evaluated by means of novel object recognition test (short- and long-term memory), inhibitory passive avoidance task (contextual learning and memory), and Morris water maze (spatial learning). In 12-month-old animals, µm‐PEA significantly improved the short-term memory in 3xTg-AD mice, with no significant effects on long-term memory. Furthermore, the compound did not exert significant effects on learning or memory in aged non-Tg mice. Interestingly, µm‐PEA also reduced depressive-like behaviors, measured by the tail suspension test and forced swim test, in early-symptomatic, but not in clearly symptomatic, 3xTg-AD mice, while it counteracted anhedonia-like phenotype of both young (6-month-old) and aging (12-month-old) 3xTg-AD mice. Overall, these findings indicate that µm‐PEA induces either beneficial cognitive or other non-cognitive effects that might be relevant to AD. Moreover, biochemical data also demonstrated that chronic µm‐PEA treatment reduced Aβ formation and phosphorylation of tau protein and promoted neuronal survival in the CA1 subregion of the hippocampus. These effects were associated with a normalization of the astrocytic function, a rebalancing of glutamatergic transmission, and a general reduction of neuroinflammatory conditions. The evidence that these biochemical/neurochemical effects were particularly manifest when the treatment was performed at a precocious stage of the pathology suggests the therapeutic potential of µm‐PEA as an early treatment in AD.

In a recent study (Bronzuoli et al., 2018), the same treatment protocol as utilized in the above research was used to evaluate the effects of the chronic µm‐PEA treatment on reactive astrogliosis and neuronal function in the frontal cortex of 6-month-old 3xTg-AD mice, compared with their age-matched non-Tg littermates. Once again, µm‐PEA demonstrated beneficial effects in reducing pathology-related biochemical alterations in this animal model of AD. In fact, 3-month µm‐PEA treatment markedly reduced astrocytic activation in 3xTg-AD mice, as demonstrated by the decrease in GFAP mRNA and protein expression and the trend toward a decrease of S100B protein expression levels. Furthermore, chronic treatment reduced iNOS levels, slightly dampened the expression of Aβ, and increased the expression of BDNF in 3xTg-AD mice. Taken together, these findings indicate that early-symptomatic 3xTg-AD mice display signs of reactive gliosis in the frontal cortex and that the chronic µm‐PEA may counteract such phenomenon, also improving the trophic support to neurons, in the absence of astrocytes and neuronal toxicity.

## Clinical Evidence

To the best of our knowledge, current clinical studies of PEA are mostly related to pain or peripheral inflammatory-related conditions, while there are very few studies aimed at evaluating the possible beneficial effects of PEA on CNS-related pathologies in human beings. This could be due to the fact that very little is known about the pharmacokinetics of PEA in humans (*please see* Pharmacokinetic). In fact, the bioavailability and apparent volume of distribution have not been clearly evaluated; and blood PEA levels, at least in animals, do not accurately reflect levels in the CNS (Davis et al., 2019). The micronized or ultramicronized forms of PEA increased bioavailability in animals compared with naïve forms, but there are very few and very recent clinical data to confirm that this is true for humans. Thus, while it seems likely that the new PEA formulations improve the compound bioavailability, complete pharmacokinetics data are urgently necessary to assess the precise tissue distribution and site of metabolism of PEA. These data will possibly allow to overcome the major difficulties in setting up clinical studies focused at evaluating the possible therapeutic role of PEA against CNS disorders.

In line with the above information, there are no clinical data concerning the possible beneficial effects of PEA in AD patients. However, Calabrò and colleagues (2016) in a case report described the case of a patient affected by mild cognitive impairment (MCI) who was treated for 9 months with high-dose PEALut. As MCI may be symptomatic of normal aging or of a transition to early AD, the results of this observation are here reported. A 67-year-old patient presented, at the onset of the observational period, a mild memory impairment, as demonstrated by the specific neuropsychological assessment, including attentive matrices (AM), Babcock Story Recall Test (BSRT), Mini-Mental State Examination (MMSE), Montreal Cognitive Assessment (MoCA), Rey Auditory Verbal Learning Test (RAVLT), Trail Making Test (TMT), and verbal fluency tests (VFTs). After a 3-month treatment with PEALut, the patient reported a non-significant cognitive amelioration, whereas her neuropsychological evaluation was almost normal after a 9-month treatment (significant improvement of RAVLT, AM, and TMT in comparison with those in the pre-treatment period).

To support the possible beneficial effect of PEA in neurodegenerative disorders, a study involving 30 Parkinson’s disease patients receiving levodopa demonstrated that uµ-PEA (600 mg for 1 year) slowed down disease progression and disability (Brotini et al., 2017).

## Conclusions

To summarize, preclinical either *in vitro* or *in vivo* data (**Tables 1** and **2**) strongly suggest that PEA, especially in its ultramicronized formulation, exerts quite robust therapeutic effects in several animal models of AD. In particular, published findings indicate that µm‐PEA treatment ameliorates both cognitive deficits and a range of neuropathological features of AD. A correlation between PEA anti-inflammatory, neuroprotective, neurobehavioral, and neurovascular effects might be suggested from the results in animal AD models, thus attributing to the compounds’ unique properties, especially compared with those of classic anti-inflammatory drugs. Despite the obvious limits of the mentioned preclinical studies and by avoiding any simplistic extrapolation of data from the animal model to the human condition, the results of these intensive preclinical experiments propose µm‐PEA as a potential therapeutic agent, which could have an impact on the progression of AD, especially when the pathology is at an early stage. This hypothesis is also supported by studies demonstrating the PEA treatment efficacy in ameliorating the symptomatology of other neurodegenerative conditions such as Parkinson’s disease (Esposito et al., 2012; Avagliano et al., 2016; Brotini et al., 2017; Crupi et al., 2018) and multiple sclerosis (Loría et al., 2008; Orefice et al., 2016).

**Table 1:** Summary of *in vitro* preclinical studies supporting the role of palmitoylethanolamide (PEA) as a possible therapeutic agent in Alzheimer’s disease (AD).

*In vitro* preclinical studies			
Preparation	Treatment	Main findings	Reference
Primary cultures of rat astrocytes	PEA (10^−7^ M) against Aβ_1–42_ (1 µg/ml)	PEA counteracts Aβ-induced reactive astrogliosis, partially through PPARα activation	Scuderi et al., 2011
Primary rat mixed neuroglial co-cultures	PEA (10^−7^ M) against Aβ_1–42_ (1 µg/ml)	PEA blunts Aβ-induced astrocyte activation and improves neuronal survival through PPARα activation	Scuderi et al., 2012; Scuderi and Steardo, 2013
Rat organotypic hippocampal slices	PEA (10^−7^ M) against Aβ_1–42_ (1 µg/ml)	PEA decreases Aβ-induced astrocyte and microglia activation, rescues neuronal CA3 damage, and reduces neuroinflammation through selective PPARα activation	Scuderi et al., 2012; Scuderi and Steardo, 2013
Primary cultures of mouse astrocytes	PEA (10^−5^ M) against Aβ_1–42_ (1 µg/ml)	PEA partially reverted the Aβ-induced inflammation	Benito et al., 2012
C6 rat astroglioma cells; HUVEC human endothelial cells	PEA (10^−8^–10^−6^ M) against Aβ_1–42_ (1 µg/ml)	PEA decreases pro-inflammatory and pro-angiogenic marker expression in Aβ-treated C6 rat astroglioma cells and in HUVEC cells exposed to the medium from Aβ-treated C6 rat astroglioma cells through PPARα activation	Cipriano et al., 2015
Primary cultures of cerebral cortex neurons and astrocytes from WT (non-Tg) and 3xTg-AD mice	PEA (10^−7^ M) against Aβ_1–42_ (0.5 µM; 24 h)	PEA prevents Aβ-induced toxicity in cultured cortical neurons and astrocytes from non-Tg mice but fails to affect the morphological alterations and glutamate levels in 3xTg-AD mouse cell cultures	Tomasini et al., 2015
Primary mouse astrocytes cell cultures and mixed astrocytes-neurons cultures	PEA (10^−7^ M) against Aβ_1–42_ (0.5 µM; 24 h)	PEA prevents Aβ-induced reduction of neuronal viability, increase of neuronal apoptotic nuclei, and decrease of MAP-2-positive neurons in astrocytes/neurons co-cultures	Beggiato et al., 2018
Primary cortical 3xTg-AD mouse astrocytes and neurons	PEA (10^−8^–10^−6^ M)	PEA reduces astrogliosis and improves neuronal viability	Bronzuoli et al., 2018
Human neurons from differentiated SH-SY5Y neuroblastoma cells	Co-ultraPEALut (2.7 and 0.27 µM) against Aβ_1–42_; (1 µM; 24 h)	Co-ultraPEALut prevents Aβ-induced reduction of cell viability and neuroinflammation	Paterniti et al., 2014
Mouse organotypic hippocampal slices	Co-ultraPEALut (2.7 and 0.27 µM) against Aβ_1–42_; (1 µM; 24 h)	Co-ultraPEALut reduces Aβ-induced iNOS, GFAP, and apoptosis and restored BDNF levels	Paterniti et al., 2014

Aβ_1–42_, β amyloid 1–42 peptide; BDNF, brain-derived neurotrophic factor; co-ultraPEALut, ultramicronized formulation of PEA/luteolin combination; GFAP, glial fibrillary acidic protein; iNOS, inducible nitric oxide synthase; MAP-2, microtubule-associated protein 2; PPARα, peroxisome proliferator-activated receptor-alpha.

**Table 2:** Summary of the available *in vivo* preclinical studies supporting the role of PEA as a possible therapeutic agent in AD.

*In vivo* preclinical studies			
Animal model	Treatment	Main findings	Reference
Mice receiving an i.c.v. injection of Aβ_25–35_ (9 nmol)	PEA (3–30 mg/kg, s.c.; starting 3 h after Aβ_25–35_, once daily for 1 or 2 weeks)	PEA, through PPARα activation, reduces/prevents Aβ_25–35_-induced behavioral impairments neuroinflammation	D’Agostino et al., 2012
Adult male rats receiving an intrahippocampal injection of Aβ_1–42_ (5 µg)	PEA (10 mg/kg; i.p., starting the day of Aβ_1–42_ injection, once daily for 1 week)	PEA prevents Aβ_1–42_–induced reactive gliosis, amyloidogenesis, tau protein hyperphosphorylation, and cognitive deficit, through PPARα activation	Scuderi et al., 2014
Young (6-month-old) and adult (12-month-old) 3xTg-AD mice	µm-PEA for 3 months (s.c. implantation of a 90-day-release pellet containing 28 mg of µm-PEA)	µm-PEA improves learning and memory, ameliorates depressive and anhedonia-like phenotype, reduces Aβ formation and phosphorylation of tau proteins, promotes neuronal survival in the CA1 subregion of the hippocampus, normalizes astrocytic function, rebalances glutamatergic transmission, and restrains neuroinflammation, especially in young early-symptomatic 3xTg-AD mice	Scuderi et al., 2018
Young (6-month-old) and adult (12-month-old) 3xTg-AD mice	µm-PEA for 3 months (s.c. implantation of a 90-day-release pellet containing 28 mg of µm-PEA)	µm-PEA reduces astrocytic activation in 3xTg-AD mice and increases the expression of BDNF in 3xTg-AD mice	Bronzuoli et al., 2018

Aβ_1–42_ = β amyloid 1–42 peptide; Aβ_25–35_ = β amyloid 25–35 peptide; BDNF, brain-derived neurotrophic factor; µm-PEA, ultramicronized PEA formulation; PPARα, peroxisome proliferator-activated receptor-alpha.

Based on the available results and for translational purposes, it becomes now urgent to evaluate the possible beneficial effects of orally administered µm‐PEA in animal models of AD. In the event of positive results, these studies would help to rapidly define adaptive clinical trials and will hopefully allow to speed up the development of an innovative therapy for AD. In this context, it is worth noting that PEA-containing products (Normast^®^, Glialia^®^, Nevamast^®^, Adolene^®^, Visimast^®^, Mastocol^®^, and Pelvilen^®^) are actually used for certain medical indications, especially inflammatory pain. Moreover, as an endogenous compound, PEA has a safely profile at pharmacological doses. Relevant PEA-induced side effects were not seen in humans at oral doses up to 1,800 mg/day. Finally, PEA has proven efficacious in humans in a number of clinical settings, and none of the clinical trials with PEA to date have reported treatment-related adverse events (Skaper et al., 2018).

## Author Contributions

LF conceptualized the review content, wrote part of the manuscript, and contributed with funding acquisition. SB conceptualized the review content and wrote part of the manuscript. MT wrote part of the manuscript and edited the final version.

## Funding

This work has been supported by a grant from Alzheimer’s Drug Discovery Foundation (ADDF) to LF (grant # 20151001) and from the University of Ferrara (FAR 2018).

## Conflict of Interest Statement

The authors declare that the research was conducted in the absence of any commercial or financial relationships that could be construed as a potential conflict of interest.

## References

[B1] Ahmad M. H. Fatima M. Mondal A. C. (2019). Influence of microglia and astrocyte activation in the neuroinflammatory pathogenesis of Alzheimer’s disease: rational insights for the therapeutic approaches. J. Clin. Neurosci. 59, 6–11. 10.1016/j.jocn.2018.10.034 30385170

[B2] Artamonov M. Zhukov O. Shuba I. Storozhuk L. Khmel T. Klimashevsky V. (2005). Incorporation of labelled *N*-acylethanolamine (NAE) into rat brain regions *in vivo* and adaptive properties of saturated NAE under X-ray irradiation. Ukr. Biokhim. Zh. 77(6), 51–62. 19618742

[B3] Aso E. Ferrer I. (2014). Cannabinoids for treatment of Alzheimer’s disease: moving toward the clinic. Front. Pharmacol. 5, 37. 10.3389/fphar.2014.00037 24634659 PMC3942876

[B4] Avagliano C. Russo R. De Caro C. Cristiano C. La Rana G. Piegari G. (2016). Palmitoylethanolamide protects mice against 6-OHDA-induced neurotoxicity and endoplasmic reticulum stress: *in vivo* and *in vitro* evidence. Pharmacol. Res. 113 (Pt A), 276–289. 10.1016/j.phrs.2016.09.004 27616549

[B5] Baker D. Pryce G. Davies W. L. Hiley C. R. (2006). In silico patent searching reveals a new cannabinoid receptor. Trends Pharmacol. Sci. 27 (1), 1–4. 10.1016/j.tips.2005.11.003 16318877

[B6] Balenga N. A. Martínez-Pinilla E. Kargl J. Schröder R. Peinhaupt M. Platzer W. (2014). Heteromerization of GPR55 and cannabinoid CB2 receptors modulates signalling. Br. J. Pharmacol. 171 (23), 5387–406. 10.1111/bph.12850 PMC4294047 25048571

[B7] Balleza-Tapia H. Crux S. Andrade-Talavera Y. Dolz-Gaiton P. Papadia D. Chen G. (2018). TrpV1 receptor activation rescues neuronal function and network gamma oscillations from Aβ-induced impairment in mouse hippocampus *in vitro*. Elife. 7, e37703. 10.7554/eLife.37703 30417826 PMC6281315

[B8] Balvers M. G. Verhoeckx K. C. Meijerink J. Wortelboer H. M. Witkamp R. F. (2013). Measurement of palmitoylethanolamide and other *N*-acylethanolamines during physiological and pathological conditions. CNS Neurol. Disord. Drug Targets 12, 23–33. 10.2174/1871527311312010007 23394528

[B9] Barbierato M. Borri M. Facci L. Zusso M. Skaper S. D. Giusti P. (2017). Expression and differential responsiveness of central nervous system glial cell populations to the acute phase protein serum amyloid A. Sci. Rep. 7 (1), 12158. 10.1038/s41598-017-12529-7 28939905 PMC5610307

[B10] Bedse G. Romano A. Lavecchia A. M. Cassano T. Gaetani S. (2015). The role of endocannabinoid signaling in the molecular mechanisms of neurodegeneration in Alzheimer’s disease. J. Alzheimers Dis. 43 (4), 1115–1136. 10.3233/JAD-141635 25147120

[B11] Beggiato S. Borelli A. C. Ferraro L. Tanganelli S. Antonelli T. Tomasini M. C. (2018). Palmitoylethanolamide blunts amyloid-β42-induced astrocyte activation and improves neuronal survival in primary mouse cortical astrocyte–neuron co-cultures. J. Alzheimers Dis. 61 (1), 389–399. 10.3233/JAD-170699 29154284

[B12] Benito C. Tolón R. M. Castillo A. I. Ruiz-Valdepeñas L. Martínez-Orgado J. A. Fernández-Sánchez F. J. (2012). β-Amyloid exacerbates inflammation in astrocytes lacking fatty acid amide hydrolase through a mechanism involving PPAR-α, PPAR-γ and TRPV1, but not CB1 or CB2 receptors. Br. J. Pharmacol. 166 (4), 1474–89. 10.1111/j.1476-5381.2012.01889.x PMC3417461 22321194

[B13] Birch A. M. Katsouri L. Sastre M. (2014). Modulation of inflammation in transgenic models of Alzheimer’s disease. J Neuroinflammation 11, 25. 10.1186/1742-2094-11-25 24490742 PMC3922595

[B14] Borrelli F. Romano B. Petrosino S. Pagano E. Capasso R. Coppola D. (2015). Palmitoylethanolamide, a naturally occurring lipid, is an orally effective intestinal anti-inflammatory agent. Br. J. Pharmacol. 172 (1), 142–58. 10.1111/bph.12907 PMC4280974 25205418

[B15] Bronzuoli M. R. Facchinetti R. Steardo L. Jr. Romano A. Stecca C. Passarella S. (2018). Palmitoylethanolamide dampens reactive astrogliosis and improves neuronal trophic support in a triple transgenic model of Alzheimer’s disease: *in vitro* and *in vivo* evidence. Oxid. Med. Cell. Longev. 2018, 4720532. 10.1155/2018/4720532 29576849 PMC5822864

[B16] Brotini S. Schievano C. Guidi L. (2017). Ultra-micronized palmitoylethanolamide: an efficacious adjuvant therapy for Parkinson’s disease. CNS Neurol. Disord. Drug Targets 16 (6), 705–713. 10.2174/1871527316666170321124949 28325153

[B17] Cai Q. Tammineni P. (2017). Mitochondrial aspects of synaptic dysfunction in Alzheimer’s disease. J. Alzheimers Dis. 57 (4), 1087–1103. 10.3233/JAD-160726 27767992 PMC5398949

[B18] Calabrò R. S. Naro A. De Luca R. Leonardi S. Russo M. Marra A. (2016). PEALut efficacy in mild cognitive impairment: evidence from a SPECT case study! Aging Clin. Exp. Res. 28 (6), 1279–1282. 10.1007/s40520-016-0533-6 26820462

[B19] Celorrio M. Rojo-Bustamante E. Fernández-Suárez D. Sáez E. Estella-Hermoso de Mendoza A. Müller C. E. (2017). GPR55: a therapeutic target for Parkinson’s disease? Neuropharmacology 125, 319–332. 10.1016/j.neuropharm.2017.08.017 28807673

[B20] Cerrato S. Brazis P. Della Valle M. F. Miolo A. Petrosino S. Di Marzo V. (2012). Effects of palmitoylethanolamide on the cutaneous allergic inflammatory response in Ascaris hypersensitive Beagle dogs. Vet. J. 191, 377–382. 10.1016/j.tvjl.2011.04.002 21601500

[B21] Chaney A. Williams S. R. Boutin H. (2018). In vivo molecular imaging of neuroinflammation in Alzheimer’s disease. J. Neurochem. 149, 438–451. 10.1111/jnc.14615 30339715 PMC6563454

[B22] Chun H. Marriott I. Lee C. J. Cho H. (2018). Elucidating the interactive roles of glia in Alzheimer’s disease using established and newly developed experimental models. Front. Neurol. 9, 797. 10.3389/fneur.2018.00797 30319529 PMC6168676

[B23] Cipriano M. Esposito G. Negro L. Capoccia E. Sarnelli G. Scuderi C. (2015). Palmitoylethanolamide regulates production of pro-angiogenic mediators in a model of β amyloid-induced astrogliosis *in vitro*. CNS Neurol. Disord. Drug Targets 14 (7), 828–837. 10.2174/1871527314666150317224155 25801844

[B24] Cristiano C. Pirozzi C. Coretti L. Cavaliere G. Lama A. Russo R. (2018). Palmitoylethanolamide counteracts autistic-like behaviours in BTBR T+tf/J mice: contribution of central and peripheral mechanisms. Brain. Behav. Immun. 74, 166–175. 10.1016/j.bbi.2018.09.003 30193877

[B25] Crupi R. Impellizzeri D. Cordaro M. Siracusa R. Casili G. Evangelista M. (2018). *N*-Palmitoylethanolamide prevents parkinsonian phenotypes in aged mice. Mol. Neurobiol. 55 (11), 8455–8472. 10.1007/s12035-018-0959-2 29552727

[B26] D’Agostino G. Russo R. Avagliano C. Cristiano C. Meli R. Calignano A. (2012). Palmitoylethanolamide protects against the amyloid-β25-35-induced learning and memory impairment in mice, an experimental model of Alzheimer disease. Neuropsychopharmacology 37 (7), 1784–1792. 10.1038/npp.2012.25 22414817 PMC3358748

[B27] Davis M. P. Behm B. Mehta Z. Fernandez C. (2019). The potential benefits of palmitoylethanolamide in palliation: a qualitative systematic review. Am. J. Hosp. Palliat. Care. 1049909119850807. 10.1177/1049909119850807 31113223

[B28] Daynes R. A. Jones D. C. (2002). Emerging roles of PPARs in inflammation and immunity. Nat. Rev. Immunol. 2 (10), 748–759. 10.1038/nri912 12360213

[B29] De Gregorio D. Manchia M. Carpiniello B. Valtorta F. Nobile M. Gobbi G. (2018). Role of palmitoylethanolamide (PEA) in depression: translational evidence. J. Affect. Disord. 255, 195–200. 10.1016/j.jad.2018.10.117 30391203

[B30] Della Valle F. Marcolongo G. Dela Valle M. F. (2013). Composition containing ultra-micronized palmitoyl-ethanolamide. United States Patent US8470373. https://patents.justia.com/patent/8470373

[B31] Devchand P. R. Keller H. Peters J. M. Vazquez M. Gonzalez F. J. Wahli W. (1996). The PPARalpha-leukotriene B4 pathway to inflammation control. Nature 384 (6604), 39–43. 10.1038/384039a0 8900274

[B32] D’Orio B. Fracassi A. Ceru M. P. Moreno S. (2018). Targeting PPARalpha in Alzheimer’s disease. Curr. Alzheimer Res. 15, 345–354. 10.2174/1567205014666170505094549 28474570

[B33] Edison P. Donat C. K. Sastre M. (2018). In vivo imaging of glial activation in Alzheimer’s disease. Front. Neurol. 9, 625. 10.3389/fneur.2018.00625 30131755 PMC6090997

[B34] Esposito E. Impellizzeri D. Mazzon E. Paterniti I. Cuzzocrea S. (2012). Neuroprotective activities of palmitoylethanolamide in an animal model of Parkinson’s disease. PLoS One 7 (8), e41880. 10.1371/journal.pone.0041880 22912680 PMC3422290

[B35] Fernández-Ruiz J. Romero J. Ramos J. A. (2015). Endocannabinoids and neurodegenerative disorders: parkinson’s disease, Huntington’s chorea, Alzheimer’s disease, and others. Handb. Exp. Pharmacol. 231, 233–259. 10.1007/978-3-319-20825-1_8 26408163

[B36] Gabrielsson L. Mattsson S. Fowler C. J. (2016). Palmitoylethanolamide for the treatment of pain: pharmacokinetics, safety and efficacy. Br. J. Clin. Pharmacol. 82 (4), 932–942. 10.1111/bcp.13020 27220803 PMC5094513

[B37] Ganley O. H. Graessle O. E. Robinson H. I. (1958). Anti-inflammatory activity on compounds obtained from egg yolk, peanut oil, and soybean lecithin. J. Lab. Clin. Med. 51 (5), 709–714. 13539486

[B38] González-Reyes R. E. Nava-Mesa M. O. Vargas-Sánchez K. Ariza-Salamanca D. Mora-Muñoz L. (2017). Involvement of astrocytes in Alzheimer’s disease from a neuroinflammatory and oxidative stress perspective. Front Mol Neurosci. 10, 427. 10.3389/fnmol.2017.00427 29311817 PMC5742194

[B39] Grillo S. L. Keereetaweep J. Grillo M. A. Chapman K. D. Koulen P. (2013). N-Palmitoylethanolamine depot injection increased its tissue levels and those of other acylethanolamide lipids. Drug Des. Devel. Ther. 7, 747–752. 10.2147/DDDT.S48324 PMC3746786 23976843

[B40] Guida F. Luongo L. Boccella S. Giordano M. E. Romano R. Bellini G. (2017). Palmitoylethanolamide induces microglia changes associated with increased migration and phagocytic activity: involvement of the CB2 receptor. Sci. Rep. 7 (1), 375. 10.1038/s41598-017-00342-1 28336953 PMC5428303

[B41] Hansen H. S. (2010). Palmitoylethanolamide and other anandamide congeners. Proposed role in the diseased brain. Exp. Neurol. 224 (1), 48–55. 10.1016/j.expneurol.2010.03.022 20353771

[B42] Heneka M. T. Carson M. J. El Khoury J. Landreth G. E. Brosseron F. Feinstein D. L. (2015). Neuroinflammation in Alzheimer’s disease. Lancet Neurol. 4 (4), 388–405. 10.1016/S1474-4422(15)70016-5 PMC5909703 25792098

[B43] Herrup K. (2010). Reimagining Alzheimer’s disease—an age-based hypothesis. J. Neurosci. 30 (50), 16755–16762. 10.1523/JNEUROSCI.4521-10.2010 21159946 PMC3004746

[B44] Hill J. D. Zuluaga-Ramirez V. Gajghate S. Winfield M. Sriram U. Rom S. (2019). Activation of GPR55 induces neuroprotection of hippocampal neurogenesis and immune responses of neural stem cells following chronic, systemic inflammation. Brain Behav. Immun. 76, 165–181. 10.1016/j.bbi.2018.11.017 30465881 PMC6398994

[B45] Holubiec M. I. Romero J. I. Suárez J. Portavella M. Fernández-Espejo E. Blanco E. (2018). Palmitoylethanolamide prevents neuroinflammation, reduces astrogliosis and preserves recognition and spatial memory following induction of neonatal anoxia-ischemia. Psychopharmacology (Berl). 235 (10), 2929–2945. 10.1007/s00213-018-4982-9 30058012

[B46] Iannotti F. A. Di Marzo V. Petrosino S. (2016). Endocannabinoids and endocannabinoid-related mediators: targets, metabolism and role in neurological disorders. Prog. Lipid. Res. 62, 107–128. 10.1016/j.plipres.2016.02.002 26965148

[B47] Impellizzeri D. Bruschetta G. Cordaro M. Crupi R. Siracusa R. Esposito E. (2014). Micronized/ultramicronized palmitoylethanolamide displays superior oral efficacy compared to nonmicronized palmitoylethanolamide in a rat model of inflammatory pain. J. Neuroinflammation 11, 136. 10.1186/s12974-014-0136-0 25164769 PMC4171547

[B48] Impellizzeri D. Siracusa R. Cordaro M. Crupi R. Peritore A. F. Gugliandolo E. (2019). *N*-Palmitoylethanolamine-oxazoline (PEA-OXA): a new therapeutic strategy to reduce neuroinflammation, oxidative stress associated to vascular dementia in an experimental model of repeated bilateral common carotid arteries occlusion. Neurobiol. Dis. 125, 77–91. 10.1016/j.nbd.2019.01.007 30660740

[B49] Inestrosa N. C. Varela-Nallar L. (2014). Wnt signaling in the nervous system and in Alzheimer’s disease. J. Mol. Cell. Biol. 6 (1), 64–74. 10.1093/jmcb/mjt051 24549157

[B50] Jayant S. Sharma B. M. Sharma B. (2016). Protective effect of transient receptor potential vanilloid subtype 1 (TRPV1) modulator, against behavioral, biochemical and structural damage in experimental models of Alzheimer’s disease. Brain Res. 1642, 397–408. 10.1016/j.brainres.2016.04.022 27084583

[B51] Jiang X. Jia L. W. Li X. H. Cheng X. S. Xie J. Z. Ma Z. W. (2013). Capsaicin ameliorates stress-induced Alzheimer’s disease-like pathological and cognitive impairments in rats. J. Alzheimers Dis. 35 (1), 91–105. 10.3233/JAD-121837 23340038

[B52] Kallendrusch S. Kremzow S. Nowicki M. Grabiec U. Winkelmann R. Benz A. (2013). The G protein-coupled receptor 55 ligand l-α-lysophosphatidylinositol exerts microglia-dependent neuroprotection after excitotoxic lesion. Glia 61 (11), 1822–1831. 10.1002/glia.22560 24038453

[B53] Kamat P. K. Kalani A. Rai S. Swarnkar S. Tota S. Nath C. (2016). Mechanism of oxidative stress and synapse dysfunction in the pathogenesis of Alzheimer’s disease: understanding the therapeutics strategies. Mol. Neurobiol. 53 (1), 648–661. 10.1007/s12035-014-9053-6 25511446 PMC4470891

[B54] Knezevic D. Mizrahi R. (2018). Molecular imaging of neuroinflammation in Alzheimer’s disease and mild cognitive impairment. Prog. Neuropsychopharmacol. Biol. Psychiatry 80 (Pt B), 123–131. 10.1016/j.pnpbp.2017.05.007 28533150

[B55] Kong W. L. Peng Y. Y. Peng B. W. (2017). Modulation of neuroinflammation: role and therapeutic potential of TRPV1 in the neuro-immune axis. Brain Behav. Immun. 64, 354–366. 10.1016/j.bbi.2017.03.007 28342781

[B56] Lambert D. M. Vandevoorde S. Diependaele G. Govaerts S. J. Robert A. R. (2001). Anticonvulsant activity of *N*-palmitoylethanolamide, a putative endocannabinoid, in mice. Epilepsia 42, 321–327. 10.1046/j.1528-1157.2001.41499.x 11442148

[B57] Levi-Montalcini R. Skaper S. D. Dal Toso R. Petrelli L. Leon A. (1996). Nerve growth factor: from neurotrophin to neurokine. Trends Neurosci. 19 (11), 514–20. 10.1016/S0166-2236(96)10058-8 8931279

[B58] Lo Verme J. Fu J. Astarita G. La Rana G. Russo R. Calignano A. (2005). The nuclear receptor peroxisome proliferator-activated receptor-alpha mediates the anti-inflammatory actions of palmitoylethanolamide. Mol. Pharmacol. 67 (1), 15–9. 10.1124/mol.104.006353 15465922

[B59] Loría F. Petrosino S. Mestre L. Spagnolo A. Correa F. Hernangómez M. (2008). Study of the regulation of the endocannabinoid system in a virus model of multiple sclerosis reveals a therapeutic effect of palmitoylethanolamide. Eur. J. Neurosci. 28 (4), 633–641. 10.1111/j.1460-9568.2008.06377.x 18657182

[B60] Martínez-Pinilla E. Reyes-Resina I. Oñatibia-Astibia A. Zamarbide M. Ricobaraza A. Navarro G. (2014). CB1 and GPR55 receptors are co-expressed and form heteromers in rat and monkey striatum. Exp. Neurol. 261, 44–52. 10.1016/j.expneurol.2014.06.017 24967683

[B61] Martínez-Pinilla E. Aguinaga D. Navarro G. Rico A. J. Oyarzábal J. Sánchez-Arias J. A. (2019). Targeting CB1 and GPR55 endocannabinoid receptors as a potential neuroprotective approach for Parkinson’s disease. Mol. Neurobiol. 10.1007/s12035-019-1495-4 30687889

[B62] Mattace Raso G. Russo R. Calignano A. Meli R. (2014). Palmitoylethanolamide in CNS health and disease. Pharmacol. Res. 86, 32–41. 10.1016/j.phrs.2014.05.006 24844438

[B63] Nam J. H. Park E. S. Won S. Y. Lee Y. A. Kim K. I. Jeong J. Y. (2015). TRPV1 on astrocytes rescues nigral dopamine neurons in Parkinson’s disease *via* CNTF. Brain 138 (Pt 12), 3610–3622. 10.1093/brain/awv297 26490328 PMC4840550

[B64] Nestmann E. R. (2016). Safety of micronized palmitoylethanolamide (microPEA): lack of toxicity and genotoxic potential. Food Sci Nutr. 5 (2), 292–309. 10.1002/fsn3.392 28265364 PMC5332261

[B65] Overk C. R. Masliah E. (2014). Pathogenesis of synaptic degeneration in Alzheimer’s disease and Lewy body disease. Biochem. Pharmacol. 88 (4), 508–516. 10.1016/j.bcp.2014.01.015 24462903 PMC3973539

[B66] Orefice N. S. Alhouayek M. Carotenuto A. Montella S. Barbato F. Comelli A. (2016). Oral palmitoylethanolamide treatment is associated with reduced cutaneous adverse effects of interferon-β1a and circulating proinflammatory cytokines in relapsing–remitting multiple sclerosis. Neurotherapeutics 13 (2), 428–438. 10.1007/s13311-016-0420-z 26857391 PMC4824021

[B67] Paterniti I. Impellizzeri D. Crupi R. Morabito R. Campolo M. Esposito E. (2013a). Molecular evidence for the involvement of PPAR-δ and PPAR-γ in anti-inflammatory and neuroprotective activities of palmitoylethanolamide after spinal cord trauma. J. Neuroinflammation 10, 20. 10.1186/1742-2094-10-20 23374874 PMC3579707

[B68] Paterniti I. Impellizzeri D. Di Paola R. Navarra M. Cuzzocrea S. Esposito E. (2013b). A new co-ultramicronized composite including palmitoylethanolamide and luteolin to prevent neuroinflammation in spinal cord injury. J. Neuroinflammation 10, 91. 10.1186/1742-2094-10-91 23880066 PMC3728012

[B69] Paterniti I. Cordaro M. Campolo M. Siracusa R. Cornelius C. Navarra M. (2014). Neuroprotection by association of palmitoylethanolamide with luteolin in experimental Alzheimer’s disease models: the control of neuroinflammation. CNS Neurol Disord Drug Targets 13 (9), 1530–1541. 10.2174/1871527313666140806124322 25106636

[B70] Pertwee R. G. (2007). GPR55: a new member of the cannabinoid receptor clan? Br. J. Pharmacol. 152 (7), 984–986. 10.1038/sj.bjp.0707464 17876300 PMC2095104

[B71] Petrosino S. Iuvone T. Di Marzo V. (2010). *N*-Palmitoyl-ethanolamine: biochemistry and new therapeutic opportunities. Biochimie 92 (6), 724–727. 10.1016/j.biochi.2010.01.006 20096327

[B72] Petrosino S. Schiano Moriello A. Cerrato S. Fusco M. Puigdemont A. De Petrocellis L. (2016). The anti-inflammatory mediator palmitoylethanolamide enhances the levels of 2-arachidonoyl-glycerol and potentiates its actions at TRPV1 cation channels. Br. J. Pharmacol. 173 (7), 1154–62. 10.1111/bph.13084 PMC5338153 25598150

[B73] Petrosino S. Di Marzo V. (2017). The pharmacology of palmitoylethanolamide and first data on the therapeutic efficacy of some of its new formulations. Br. J. Pharmacol. 174 (11), 1349–1365. 10.1111/bph.13580 27539936 PMC5429331

[B74] Petrosino S. Cordaro M. Verde R. Schiano Moriello A. Marcolongo G. Schievano C. (2018). Oral ultramicronized palmitoylethanolamide: plasma and tissue levels and spinal anti-hyperalgesic effect. Front. Pharmacol. 9, 249. 10.3389/fphar.2018.00249 29615912 PMC5870042

[B75] Puglia C. Blasi P. Ostacolo C. Sommella E. Bucolo C. Platania C. B. M. (2018). Innovative nanoparticles enhance *N*-palmitoylethanolamide intraocular delivery. Front. Pharmacol. 9, 285. 10.3389/fphar.2018.00285 29643808 PMC5882782

[B76] Rinne P. Guillamat-Prats R. Rami M. Bindila L. Ring L. Lyytikäinen L. P. (2018). Palmitoylethanolamide promotes a proresolving macrophage phenotype and attenuates atherosclerotic plaque formation. Arterioscler. Thromb. Vasc. Biol. 38 (11), 2562–2575. 10.1161/ATVBAHA.118.311185 30354245

[B77] Ross R. A. (2003). Anandamide and vanilloid TRPV1 receptors. Br. J. Pharmacol. 140 (5), 790–801. 10.1038/sj.bjp.0705467 14517174 PMC1574087

[B78] Saito T. Saido T. C. (2018). Neuroinflammation in mouse models of Alzheimer’s disease. Clin. Exp. Neuroimmunol. 9 (4), 211–218. 10.1111/cen3.12475 30546389 PMC6282739

[B79] Saliba S. W. Jauch H. Gargouri B. Keil A. Hurrle T. Volz N. (2018). Anti-neuroinflammatory effects of GPR55 antagonists in LPS-activated primary microglial cells. J. Neuroinflammation 15 (1), 322. 10.1186/s12974-018-1362-7 30453998 PMC6240959

[B80] Scuderi C. Esposito G. Blasio A. Valenza M. Arietti P. Steardo L. Jr. (2011). Palmitoylethanolamide counteracts reactive astrogliosis induced by β-amyloid peptide. J. Cell. Mol. Med. 15 (12), 2664–2674. 10.1111/j.1582-4934.2011.01267.x 21255263 PMC4373435

[B81] Scuderi C. Valenza M. Stecca C. Esposito G. Carratù M. R. Steardo L. (2012). Palmitoylethanolamide exerts neuroprotective effects in mixed neuroglial cultures and organotypic hippocampal slices *via* peroxisome proliferator-activated receptor-α. J. Neuroinflammation 9, 49. 10.1186/1742-2094-9-21 22405189 PMC3315437

[B82] Scuderi C. Steardo L. (2013). Neuroglial roots of neurodegenerative diseases: therapeutic potential of palmitoylethanolamide in models of Alzheimer’s disease. CNS Neurol. Disord. Drug Targets 12 (1), 62–9. 10.2174/1871527311312010011 23394526

[B83] Scuderi C. Stecca C. Valenza M. Ratano P. Bronzuoli M. R. Bartoli S. (2014). Palmitoylethanolamide controls reactive gliosis and exerts neuroprotective functions in a rat model of Alzheimer’s disease. Cell. Death Dis. 5, e1419. 10.1038/cddis.2014.376 25210802 PMC4540191

[B84] Scuderi C. Bronzuoli M. R. Facchinetti R. Pace L. Ferraro L. Broad K. D. (2018). Ultramicronized palmitoylethanolamide rescues learning and memory impairments in a triple transgenic mouse model of Alzheimer’s disease by exerting anti-inflammatory and neuroprotective effects. Transl. Psychiatry 8 (1), 32. 10.1038/s41398-017-0076-4 29382825 PMC5802581

[B85] Skaper S. D. Facci L. Giusti P. (2014). Mast cells, glia and neuroinflammation: partners in crime? Immunology 141 (3), 314–327. 10.1111/imm.12170 24032675 PMC3930370

[B86] Skaper S. D. Facci L. Zusso M. Giusti P. (2018). An inflammation-centric view of neurological disease: beyond the neuron. Front. Cell. Neurosci. 12, 72. 10.3389/fncel.2018.00072 29618972 PMC5871676

[B87] Stampanoni Bassi M. Gentile A. Iezzi E. Zagaglia S. Musella A. Simonelli I. (2019). Transient receptor potential vanilloid 1 modulates central inflammation in multiple sclerosis. Front. Neurol. 10, 30. 10.3389/fneur.2019.00030 30761069 PMC6361812

[B88] Stančić A. Jandl K. Hasenöhrl C. Reichmann F. Marsche G. Schuligoi R. (2015). The GPR55 antagonist CID16020046 protects against intestinal inflammation. Neurogastroenterol. Motil. 27 (10), 1432–1445. 10.1111/nmo.12639 26227635 PMC4587547

[B89] Steardo L. Jr. Bronzuoli M. R. Iacomino A. Esposito G. Steardo L. Scuderi C. (2015). Does neuroinflammation turn on the flame in Alzheimer’s disease? Focus on astrocytes. Front. Neurosci. 9, 259. 10.3389/fnins.2015.00259 26283900 PMC4518161

[B90] Straus D. S. Glass C. K. (2007). Anti-inflammatory actions of PPAR ligands: new insights on cellular and molecular mechanisms. Trends Immunol. 28 (12), 551–558. 10.1016/j.it.2007.09.003 17981503

[B91] Tomasini M. C. Borelli A. C. Beggiato S. Ferraro L. Cassano T. Tanganelli S. (2015). Differential effects of palmitoylethanolamide against amyloid-β induced toxicity in cortical neuronal and astrocytic primary cultures from wild-type and 3xTg-AD mice. J. Alzheimers Dis. 46 (2), 407–421. 10.3233/JAD-143039 25765918

[B92] Tronino D. Russo R. Ostacolo C. Mazzolari A. De Caro C. Avagliano C. (2015). Improvement of topical palmitoylethanolamide anti-inflammatory activity by pegylated prodrugs. Mol. Pharm. 12, 3369–3379. 10.1021/acs.molpharmaceut.5b00397 26289562

[B93] Tsuboi K. Ikematsu N. Uyama T. Deutsch D. G. Tokumura A. Ueda N. (2013). Biosynthetic pathways of bioactive *N*-acylethanolamines in brain. CNS Neurol. Disord. Drug Targets 12 (1), 7–16. 10.2174/1871527311312010005 23394527

[B94] Tsuboi K. Uyama T. Okamoto Y. Ueda N. (2018). Endocannabinoids and related *N*-acylethanolamines: biological activities and metabolism. Inflamm. Regen. 38, 28. 10.1186/s41232-018-0086-5 30288203 PMC6166290

[B95] Vacondio F. Bassi M. Silva C. Castelli R. Carmi C. Scalvini L. (2015). Amino acid derivatives as palmitoylethanolamide prodrugs: synthesis, *in vitro* metabolism and *in vivo* plasma profile in rats. PLoS One 10 (6), e0128699. 10.1371/journal.pone.0128699 26053855 PMC4460047

[B96] Vale C. Alonso E. Rubiolo J. A. Vieytes M. R. LaFerla F. M. Giménez-Llort L. (2010). Profile for amyloid-beta and tau expression in primary cortical cultures from 3xTg-AD mice. Cell. Mol. Neurobiol. 30 (4), 577–590. 10.1007/s10571-009-9482-3 19943189 PMC11498757

[B97] Van Eldik L. J. Carrillo M. C. Cole P. E. Feuerbach D. Greenberg B. D. Hendrix J. A. (2016). The roles of inflammation and immune mechanisms in Alzheimer’s disease. Alzheimers Dement (NY). 2 (2), 99–109. 10.1016/j.trci.2016.05.001 PMC5644267 29067297

[B98] Wirz K. T. Keitel S. Swaab D. F. Verhaagen J. Bossers K. (2014). Early molecular changes in Alzheimer disease: can we catch the disease in its presymptomatic phase? J. Alzheimers Dis. 38 (4), 719–740. 10.3233/JAD-130920 24072070

[B99] Xu W. Liu J. Ma D. Yuan G. Lu Y. Yang Y. (2017). Capsaicin reduces Alzheimer-associated tau changes in the hippocampus of type 2 diabetes rats. PLoS One 12 (2), e0172477. 10.1371/journal.pone.0172477 28225806 PMC5321461

[B100] Yang H. Zhou J. Lehmann C. (2016). GPR55—a putative “type 3” cannabinoid receptor in inflammation. J. Basic Clin. Physiol. Pharmacol. 27 (3), 297–302. 10.1515/jbcpp-2015-0080 26669245

[B101] Zádori D. Veres G. Szalárdy L. Klivényi P. Vécsei L. (2018). Alzheimer’s disease: recent concepts on the relation of mitochondrial disturbances, excitotoxicity, neuroinflammation, and kynurenines. J. Alzheimers Dis. 62 (2), 523–547. 10.3233/JAD-170929 29480191

[B102] Zhao Z. Wang J. Wang L. Yao X. Liu Y. Li Y. (2017). Capsaicin protects against oxidative insults and alleviates behavioral deficits in rats with 6-OHDA-induced Parkinson’s disease *via* activation of TRPV1. Neurochem. Res. 42 (12), 3431–3438. 10.1007/s11064-017-2388-4 28861768

[B103] Zhukov O. D. (1999). Distribution of *N*-([1-^14^C]-palmitoyl)ethanolamine in rat tissues. Ukr. Biokhim. Zh. 71 (4), 124–125. 10791074

[B104] Zimmer E. R. Leuzy A. Benedet A. L. Breitner J. Gauthier S. Rosa-Neto P. (2014). Tracking neuroinflammation in Alzheimer’s disease: the role of positron emission tomography imaging. J. Neuroinflammation 11, 120. 10.1186/1742-2094-11-120 25005532 PMC4099095

[B105] Zygmunt P. M. Ermund A. Movahed P. Andersson D. A. Simonsen C. Jönsson B. A. (2013). Monoacylglycerols activate TRPV1—a link between phospholipase C and TRPV1. PLoS One 8 (12), e81618. 10.1371/journal.pone.0081618 24312564 PMC3847081

